# Asynchronous dentofacial development and dental crowding: a cross-sectional study in a contemporary sample of children in France

**DOI:** 10.1186/1880-6805-32-22

**Published:** 2013-11-19

**Authors:** Wei Yan-Vergnes, Jean-Noel Vergnes, Jean Dumoncel, Pascal Baron, Christine Marchal-Sixou, José Braga

**Affiliations:** 1 Department of Orthodontics, Toulouse Dental Faculty, Paul Sabatier University, Faculté de Chirurgie Dentaire, (3 chemin des maraîchers), Toulouse Cedex 04 31062, France; 2 Department of Epidemiology, Toulouse Dental Faculty and Toulouse University Hospital, Paul Sabatier University, Faculté de Chirurgie Dentaire, (3 chemin des maraîchers), Toulouse Cedex 04 31062, France; 3 AMIS Laboratory, UMR 5288, Université Toulouse IIII/CNRS/ Faculté de médecine, (37 allées J. Guesdes), Toulouse Cedex 3 31073, France

## Abstract

**Background:**

The causes of dental crowding are not fully understood, but it may result from an evolutionary trend towards reduced facial volume, without a proportional reduction in tooth sizes. Most previous studies conducted among modern humans have revealed a very low or non-existent correlation between tooth size and jaw size. Cross-comparison between dental age and facial skeletal age could help to provide better knowledge of the dynamic process of dental crowding. The primary objective of this research was to study the synchronism of dental maturation and skeletal facial growth in a sample of modern children living in France. The secondary objective was to assess the link between dentofacial asynchronism and dental crowding.

**Results:**

The random sample comprised 28 subjects (16 girls, 12 boys). Mean chronological age was 13.5 years (±2.1; range 9.2–17.6). Mean dental age was 14.2 years (±2.8; range 7.5–17) and mean facial skeletal age was 12.8 years (±2.6, range 7–22). In the estimations of dental age and facial skeletal age, there was no evidence of systematic bias. There were 10 subjects (9 girls, 1 boy) with asynchronous dentofacial development. Finally, there were 13 subjects (8 girls, 5 boys) with dental crowding. A significant association was found between delayed facial skeletal growth/advanced dental maturation and dental crowding (*P* = 0.01).

**Conclusions:**

Dental maturation and facial growth are not necessarily synchronous. Further understanding of the interactions between dental maturation and facial growth could have crucial implications in biological anthropology, as well as for the clinical practice of orthodontists. From an anthropological perspective, this study suggests that asynchronous dentofacial development could, at least partially, explain the frequency of dental crowding in modern populations.

## Background

Research in physiological anthropology is essential to a better understanding of past and present human populations. In facial anthropology, the study of the timing of facial growth and its relationship to dental maturation is of special interest for the purpose of improving knowledge about human evolution (paleoanthropology) [1], for medical purposes (orthodontic care) [2] and for legal purposes (age estimation in forensic anthropology) [3]. A condition related to both facial development and dental maturation is dental crowding.

Dental crowding can be defined as a discrepancy between tooth and jaw sizes that results in malposition and/or rotation of teeth [4]. Dental crowding is not a disease in itself but can be considered a condition that can lead to or promote diseases [5] such as periodontal disease [6], dental caries [7] or temporomandibular joint dysfunction [8]. Nowadays, the prevalence of dental crowding is estimated to range between 30% and 60% [9-12]. It is one of the most frequent reasons why people consult an orthodontist, especially given the high aesthetic demand expressed by patients [13].

The causes of dental crowding are still not fully understood, but they may rooted in an evolutionary trend toward reduced facial volume without a proportional reduction in tooth size [14]. Most previous studies conducted among primates have revealed a very low or nonexistent correlation between tooth size and jaw size [15,16]. It has been shown that, as human populations transitioned from a hunter-gatherer lifestyle to an agricultural one, there was a consistent shift toward a shorter and broader mandible [17]. Thus, dental crowding could occur as a result of the increased processing of modern foods and thus a reduced need for powerful masticatory action [18].

Independently of the causes of dental crowding, several studies have examined the extent to which tooth and jaw sizes contribute to the condition. It has been found that groups of individuals with dental crowding have smaller dental arch dimensions than noncrowded groups [4,19]. McKeown [20] found a stronger correlation between arch size and dental crowding than between tooth size and dental crowding. These observations are controversial, however, and varying numbers of confirming and refuting observations are available. For example, a recent study suggested that tooth size could play a greater role than jaw size in the development of dental crowding [21].

To the best of our knowledge, most previous studies that have assessed discrepancies between tooth size and jaw size have used raw clinical and/or cephalometric measurements without focusing on the physiological stages of dental maturation or stages of facial growth. Stages of dental maturation in humans have been the subject of extensive research, and Demirjian *et al*.’s method [22] for age estimation based on teeth is now widely used in both anthropology and forensics [23-26]. More recently, a method was devised to estimate the age of children by using the centroid size of their facial skeleton [27].

Cross-comparison between dental age and facial skeletal age could help to provide better knowledge of the dynamic process of dental crowding. Our hypothesis is that, at the individual level, dental crowding could be the result of an asynchronism between dental maturation and facial growth.

The primary aim of our present study was to examine the synchronism of dental maturation and facial growth in a sample of modern children. The secondary aim was to assess the link between dentofacial asynchronism and dental crowding.

## Methods

### Study design and sampling methods

We conducted a retrospective cross-sectional study based on computed tomography (CT) scans of a contemporaneous cohort of children of various origins who lived in the Toulouse, France, area. The sample was drawn from a database of CT scans taken between 2001 and 2010 in the Neuroradiology Unit of the Clinique Pasteur, Toulouse, and Toulouse University Hospital. CT scans of the children had been taken because of trauma, impacted or residual teeth, inflammation in the maxillary sinuses or neonatal distress. The whole database is securely hosted in the Anthropology Laboratory of Molecular Imaging and Synthesis (UMR5288, CNRS). A French institutional review board provided ethical approval of the study [27]. The CT data were anonymous and numbered. Each number was entered into an Excel file with, exclusively, the date of birth, the date of the CT scan and the child’s sex.

To be included in the present analysis, participants had to be younger than 18 years of age, have all their mandibular permanent teeth (erupted or not, except the wisdom teeth), have a Demirjian stage [22] of at least 6 for mandibular permanent incisors and at least 7 for the first mandibular permanent molars (to allow for space analysis using Nance’s principles [28]) and have complete radiographic acquisition to permit assessment of dental age, facial skeletal age and dental crowding.

From the initial database, sampling was performed in two phases. Participants were first randomly selected from the initial database, then inclusion criteria were applied to incrementally produce the working database until the sample size calculated *a priori* was attained (see Statistical considerations).

### Data

Facial skeletal age was estimated using a method previously developed to assess the skeletal age of children, based on the centroid size of the face and derived from the three-dimensional coordinates of anatomical landmarks [27]. The anatomical locations of bilateral landmarks (mental foramen, anterior opening of the infraorbital canal, anterior opening of the supraorbital canal, supraorbital fissure at the level of the optic foramen and round foramina) were selected using AMIRA 3D analysis software (version 5.2.2; FEI Visualization Sciences Group, Mérignac, France), and centroid sizes were calculated using morphologika software [29]. The centroid size is a measure of size uncorrelated with all pure shape changes [30]. The facial skeletal age was calculated using least squares linear regression (with distinction between boys and girls and between children younger or older than 10 years) [27]. Chronological and facial skeletal ages were rounded down to the nearest semester. A child was considered as presenting delayed facial skeletal growth if his or her chronological age was strictly greater than the estimation of his or her facial skeletal age (that is, greater than and not equal to).

Dental age was estimated by using a previously described method [25]. A panoramic projection of teeth from Digital Imaging and Communications in Medicine, or DICOM, files allowed Demirjian’s stages [22] of the right hemimandible of each child [31] to be assessed. Estimation of dental age was based on Bayesian predictions [25]. Chronological and dental ages were rounded down to the nearest semester. A child was considered as presenting advanced dental maturation if his or her chronological age was strictly less than the estimation of dental age.

Regarding synchronism, a child was considered as revealing asynchronous dentofacial development if he or she had (1) delayed facial skeletal growth and advanced dental maturation (DS/AD) or (2) advanced facial skeletal growth and delayed dental maturation (AS/DD). Thus, we distinguished two types of asynchronous dentofacial development: the DS/AD type and the AS/DD type. By definition, we considered all other children as having synchronous dentofacial development.

Space analysis required a comparison between the amount of space actually available for the alignment of the teeth and the amount of space necessary to align them properly [32]. Space conditions were calculated following Nance’s principles [28] using OsiriX imaging software (version 4.0; http://www.osirix-viewer.com/). Individual mesiodistal crown lengths of all mandibular permanent teeth, exclusive of molars, were measured. The cumulative mesiodistal crown lengths defined the space required. Mandibular arch perimeter was measured from the mesial surface of the first permanent molar to the mesial surface of the opposite molar, thus defining the available space. Next, the difference between the space available and the space required was calculated (expressed in millimeters). A child was considered to have dental crowding if the space required was strictly greater than the space available.

### Statistical considerations

The null hypothesis was that the proportion of children with dental crowding was the same in both groups, with or without DS/AD asynchronous dentofacial development.

Sample size was calculated using the normal approximation to the arcsine transformation of the binomial distribution [33]. The sample size required to detect a difference in proportion of 50% between the two groups at the significance level of 5% with a power of 80% is 28. Groups were compared with a two-sided Fisher’s exact test.

Statistical procedures for estimating dental age and facial skeletal age have been detailed in previous articles [25,27]. As described in these articles, estimation of dental age and facial skeletal age were performed using different algorithms for boys and girls.

Dental age and facial skeletal age were compared with chronological age using the Bland–Altman plotting method [34]. In this method, the differences between the age estimate and the chronological age are plotted against the averages of age estimates and chronological ages. We used the Bland–Altman plot to investigate any possible relationship of the discrepancies between age estimations and chronological age (that is, proportional bias). Proportional bias occurs when age estimation and chronological age do not agree equally throughout the range of measurements. Horizontal lines were drawn at the mean difference and at the limits of agreement (mean difference ±1.96). The software packages used were Scilab software (version 5.3.3; Scilab Enterprises, Versailles, France) for estimating dental age using the Bayesian approach and R version 2.15.1 software (The R Project for Statistical Computing; http://www.r-project.org/) for all statistical analyses.

## Results

### Visualization of cases

**Figure 1:**
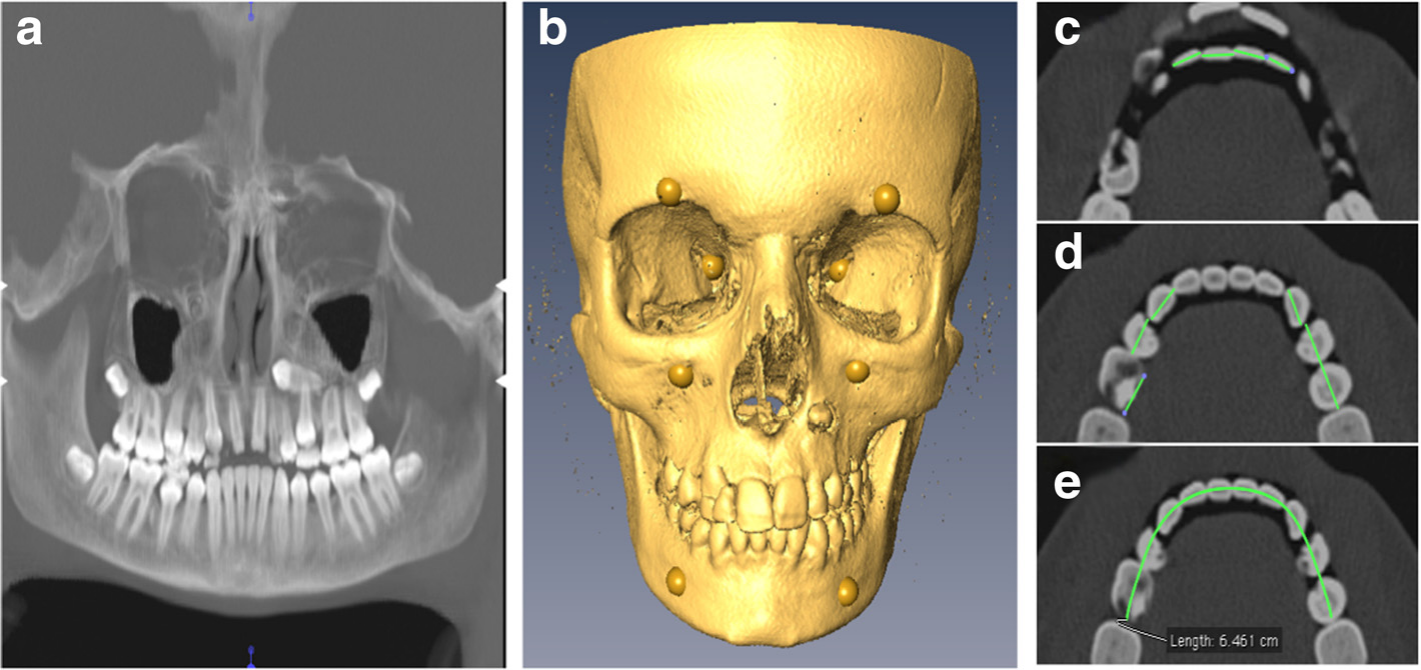
**Images of girl, 13.0 years of age, with synchronous dentofacial development without dental crowding. (a)** Panoramic projection of teeth. Dental age = 12.5 years. **(b)** Anatomical landmarks used for calculation of the centroid size. Facial skeletal age = 13.0 years. Note the horizontal impaction of the upper left canine in **(a)** and **(b)**. Synchronous dentofacial development, even without mandibular dental crowding, does not mean absence of any other stomatognathic system abnormalities. **(c)** Individual mesiodistal crown lengths of incisors (W_i_). Measurement of mesiodistal crown lengths of mandibular permanent teeth implies multiple horizontal computed tomographic scan slices. As images were obtained in a closed mouth position, horizontal section images could reveal both mandibular and maxillary tooth structures. **(d)** Individual mesiodistal crown lengths of canines and premolars (W_cp_). W_i_ + W_cp_ = 63.0 mm. **(e)** Arch perimeter = 64.6 mm. Green lines in **(c)** and **(d)** are mesiodistal crown lengths. Green curve in **(e)** is the arch perimeter (blue dots are construction points).

**Figure 2:**
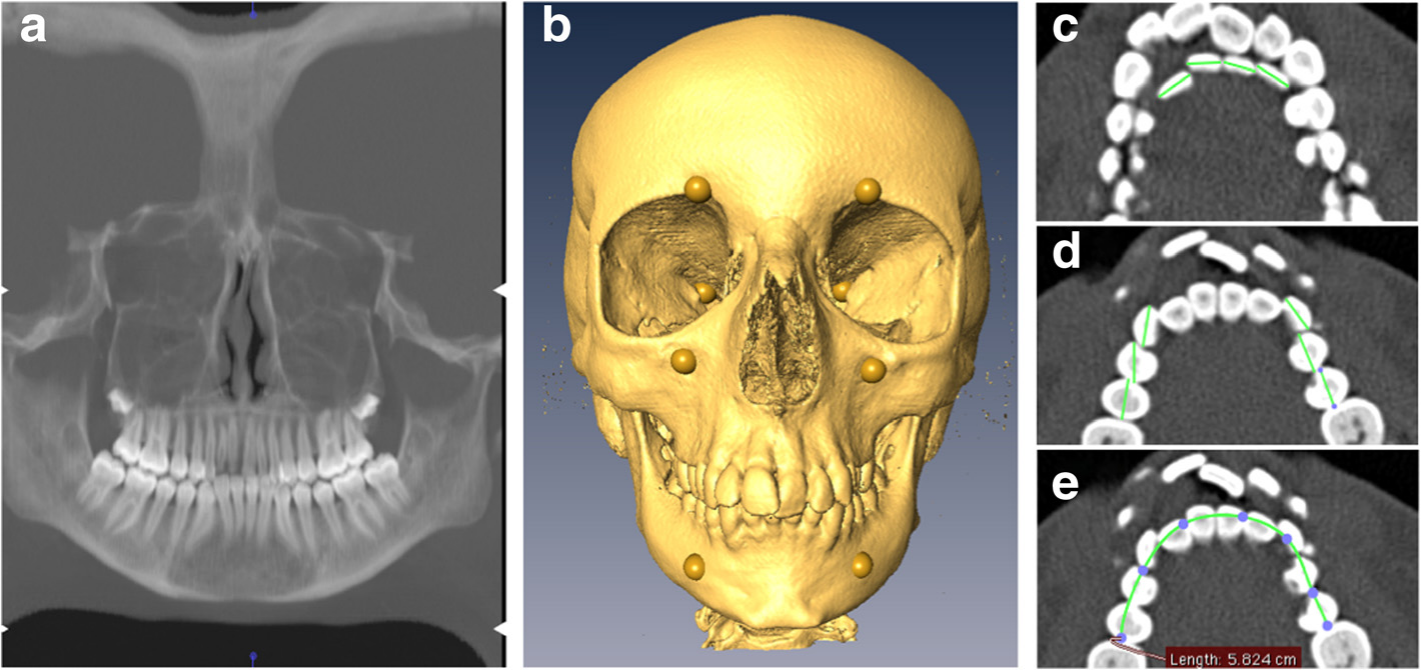
**Images of girl, 13.5 years of age, with delayed facial skeletal growth and advanced dental maturation asynchronous dentofacial development and dental crowding. (a)** Panoramic projection of teeth. Dental age = 16.2. **(b)** Anatomical landmarks used to calculate the centroid size. Facial skeletal age = 12.0. **(c)** Individual mesiodistal crown lengths of incisors (W_i_). **(d)** Individual mesiodistal crown lengths of canines and premolars (W_cp_). W_i_ + W_cp_ = 68.0 mm. **(e)** Arch perimeter = 58.2 mm. In **(c)**, **(d)** and **(e)**, measurement of mesiodistal crown lengths of mandibular permanent teeth implies multiple horizontal computed tomographic scan slices. As images were obtained with the children’s mouths closed, horizontal section images could reveal both mandibular and maxillary tooth structures. Green lines in **(c)** and **(d)** are mesiodistal crown lengths. Green curve in **(e)** is the arch perimeter (blue dots are construction points).

Figure 1 shows an example of a child with synchronous dentofacial development without dental crowding. In contrast, Figure 2 presents an example of a child with DS/AD asynchronous dentofacial development and dental crowding.

### Descriptive analysis of the sample

**Figure 3:**
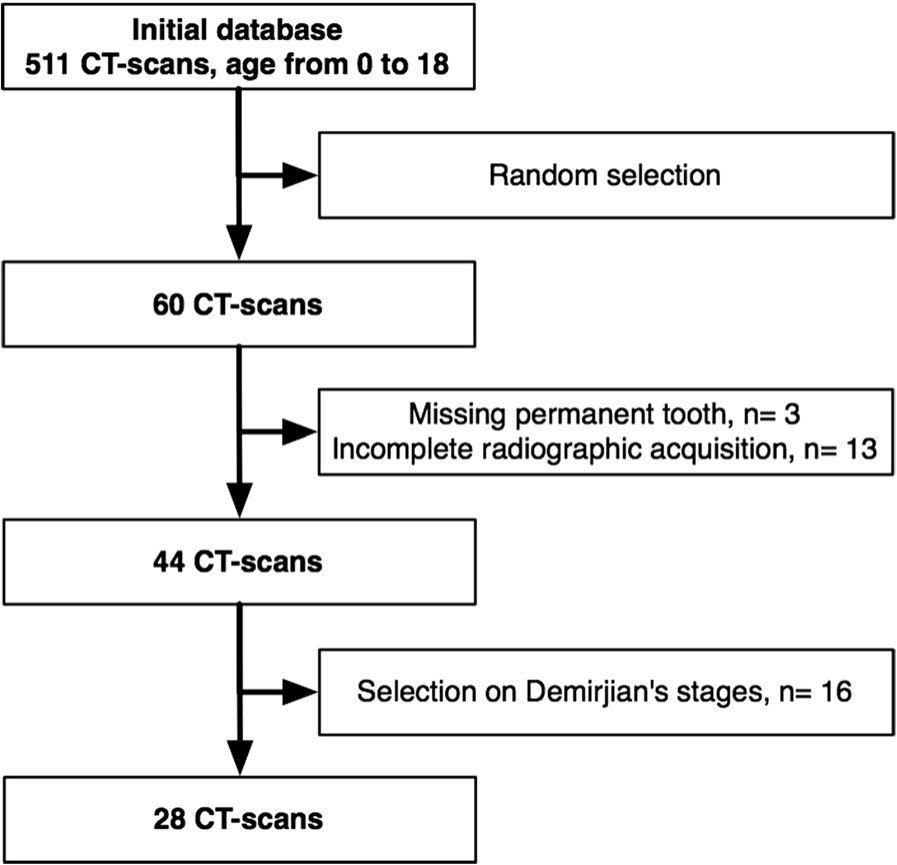
**Flow diagram of the sampling process.** CT, computed tomography.

The random sample of 28 children was made up of 16 girls and 12 boys. A flow diagram of the sampling process is presented in Figure 3. The children’s mean chronological age was 13.5 years (±2.1; range = 9.2 to 17.6). Mean dental age was 14.2 years (±2.8; range = 7.5 to 17), and mean facial skeletal age was 12.8 years (±2.6; range = 7 to 22).

**Figure 4:**
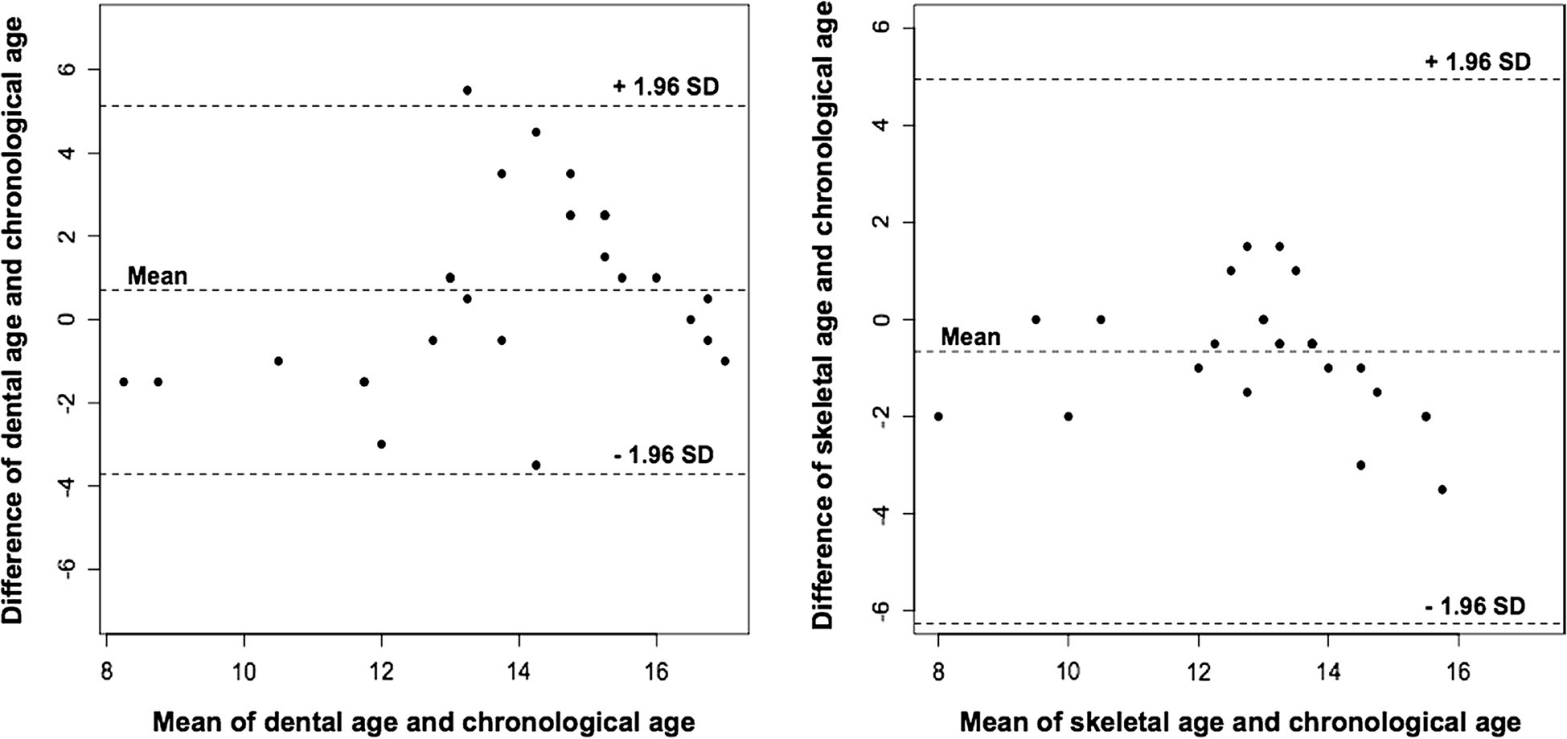
**Bland–Altman plot between age estimations and chronological age.** Because of the discrete values of Bayesian estimates, the values of ages are not continuous. This is why some points are superimposed.

In the estimations of dental age and facial skeletal age using the Bland–Altman method, there was no evidence of proportional bias. The whole sample was generally located between the upper and lower limits of agreement (mean ± 1.96 SD), with a slight overestimation for dental age (line of the mean above zero) and a slight underestimation for facial skeletal age (line of the mean below zero) relative to chronological age (Figure 4).

There were 10 children (9 girls and 1 boy) with DS/AD asynchronous dentofacial development, and only one (a boy) with AS/DD asynchronous dentofacial development. There were 13 children (8 girls and 5 boys) with dental crowding.

### Comparative analysis

Table 1 shows the 2 × 2 cross-tabulation of the presence or absence of DS/AD asynchronous dentofacial development against the presence or absence of dental crowding. There was a significant association between DS/AD and dental crowding (*P* = 0.01). DS/AD children were more prone to dental crowding. In contrast, the only AS/DD child did not have dental crowding.

**Table 1:** **Cross-tabulation of the presence or absence of delayed facial skeletal growth and advanced dental maturation asynchronous dentofacial development**^**a **^**against the presence or absence of dental crowding**

		**DS/AD asynchronous dentofacial development**		
		**Yes**	**No**	**Total**
Dental crowding	Yes	8	5	13
	No	2	13	15
Total		10	18	28

^a^DS/AD, delayed facial skeletal growth and advanced dental maturation.

## Discussion

The results of this study indicate that dental maturation and facial growth are not necessarily synchronous. In our present study, we found a significant association between DS/AD and dental crowding (*P* = 0.01). Although an observed association does not imply causation [35], this result is concordant with the existing body of studies in which researchers investigated the possible etiology of dental crowding. More precisely, our study favors the theory that dental crowding could be a consequence of low masticatory stresses, not only at the human evolution level but also at the individual level. This theory is congruent with the hypothesis that facial shape differences observed between agricultural and hunter-gatherer populations are significantly attributable to phenotypic plasticity rather than to natural selection only [17]. Carlson and Van Gerven argued that changes in masticatory function and diet had so reduced chewing stress that the jaws did not develop to a sufficient size to hold all the teeth [36]. In previous localized comparisons of hunter-gatherer and farming populations, significant changes occurring in the masticatory apparatus were found to occur within a relatively short time period, suggesting developmental plasticity or rapid selection. The delayed facial skeletal growth observed in some individuals in our study sample could be related to inadequate chewing stresses, thus generating insufficient strain for mandibular growth in relation to overall tooth size [37]. This could lead to reduced alveolar and corpus lengths, as well as reduced skeletal facial volume, thus diminishing the space for complete dental eruption [17]. Several arguments published in the literature can be put forward to support this interpretation of our results. For example, a recent study showed that there was an association between mandibular size and number of molars retained in occlusion, with smaller mandibular dimensions found in individuals retaining fewer occluding molars [38]. A study of nonhuman primates found that hyraxes raised on cooked food had significantly less growth (approximately 10%) in the ventral (inferior) and posterior portions of the face [39]. Although some authors have suggested that dental crowding is more frequent in modern populations than among ancient populations [40], diachronic changes in archaeological settings have been reported only minimally [41]. In fact, evidence from the eastern Mediterranean, Southeast Asia and the American Southeast indicates that increased dental crowding rates coincide with a shift to agricultural societies and softer, westernized diets [41].

Rose and Roblee favored the explanation that reduced chewing stress in childhood produced jaws that were too small for the teeth, despite the ubiquitous trend in tooth size reduction [42]. Kelley and Larsen suggested a strong association of alveolar bone growth with the functional stimulation of chewing forces. This work included measurements of bite-force variation between generations of Eskimos and animal studies showing changes in mandibular growth of rats and primates between groups that ate hard or soft diets [43]. Diet-associated reduction in chewing stress was found to result in decreased growth of the mandibular and maxillary arches and, in animal studies, in both facial reduction and increased malocclusion in the low-force groups [42].

These current explanations contrast with Begg’s suggestions, published in the mid-1950s, that interstitial attrition caused by chewing forces reduced the mesiodistal lengths of all teeth so that they could fit within the jaw [44]. This hypothesis has always failed to explain early incisor crowding as well as crowding in children and adolescents prior to an age when attrition becomes advanced [40]. However, some researchers today still maintain that dental crowding might be genetic in origin and might not be caused by excessive tooth size or changes in environmental factors (masticatory activity) [45]. Normando and colleagues recently emphasized the role of heredity in the occurrence of dental crowding [46], suggesting a highly polygenic basis for complex traits such as human craniofacial and dentition morphology and development [47].

An unexpected finding of our present study is that DS/AD asynchronous dentofacial development was more frequent among girls than among boys (relative ratio: (9/16)/(1/12) ≈ 7). Moreover, the only case of AS/DD asynchronous dentofacial development was found in a boy.

This finding could not be attributed to the well-documented systematic differences between boys and girls regarding the timing of dental or skeletal maturation, because age estimations were performed separately on each sex subset within the database, in accordance with the methods we used. Several explanations could be suggested. First, our study was designed primarily to assess the link between dentofacial asynchronism and dental crowding. This unexpected finding could be explained by a study design inadequate to test the hypothesis of a difference in AS/DD asynchronism between girls and boys. Second, we cannot reject a negative potentiation between delayed facial skeletal growth and advanced dental maturation among girls or a positive potentiation among boys. The reasons for these putative synergistic effects remain unclear, but they could be related to sexual dimorphism. Sexual dimorphism of the adult modern human facial skeleton, including the mandible, has been noted in diverse populations [48]. Further studies are required to search for the link between sex and dentofacial asynchronism.

Our findings could have practical clinical implications. Knowledge of the facial skeletal growth and dental maturation status could be a useful tool in orthodontic treatment planning. Further development of tools for treatment planning could allow the orthodontist to integrate the assessment of dentofacial asynchronism into daily decision-making. For example, severe dental crowding in a child with DS/AD is not likely to lead to the same treatment plan as dental crowding in a child of the same age with AS/DD. In the first case, growth stimulation should be considered, whereas in the second, serial extraction or dental stripping might be a better option. Prediction of facial growth velocity and percentage of facial growth remaining can be important in orthodontic treatment planning [49] and may be essential if the purpose of the prediction is to take advantage of it during orthodontic treatment [50], especially in the presence of dental crowding. An accurate prediction of the pubertal growth spurt might be beneficial in the treatment of some types of malocclusion associated with skeletal disorders [51]. In some cases, less tooth movement may be required, and growth may be an ally; under other clinical conditions, tooth movement will have a predominant role, depending on whether the growth pattern is favorable or unfavorable [50]. All individuals undergo a pubertal growth spurt, but there are noteworthy differences in onset, duration, velocity and amount of growth [50]. Although a significant correlation has been found between skeletal maturation and facial growth spurt at the population level [52], it is well-known that the bones of the face are formed by intramembranous ossification without cartilaginous precursors. Thus, growth of the face may be regulated by factors other than those responsible for growth of the long bones [49]. It remains challenging to personalize orthodontic treatment-planning at the individual level, because genetic factors and differences between sex and ethnic group are also associated with the onset, duration, intensity and end of the growth spurt [50]. All of these factors are likely to interact with each other and have differential effects on facial growth, statural growth or biological maturation. Moreover, the findings of our present study could support the hypothesis that dental maturation may not be linked directly to facial or mandibular growth [53]. Again, despite a significant correlation between dental maturation and bone age found in many studies of various populations [54-56], such correlations are generally considered to be moderate at best [57]. This could explain why dental development indicators are not reliable predictors of an individual’s stage of skeletal development.

The present study has several limitations. We considered the chronological age as the reference for estimating both dental age and facial skeletal age. The concepts of “advanced” and “delayed” maturation are relative to the mean values of the population from which the estimation methods have been developed. The population analyzed in our study comprised a random sample of the database used to generate the method for estimating facial skeletal age. Both methods used in this analysis are freely available [25,27] and need to be studied in other populations. Additionally, it might be of special interest to analyze the factors associated with the occurrence of dental crowding among people with asynchronous dentofacial development. A longitudinal study could be conducted with analysis using a life-course approach, taking into account the determinants of dental crowding from a biopsychosocial perspective.

## Conclusions

Dental maturation and facial growth are not necessarily synchronous. At the individual level, in the presence of dental crowding, orthodontic treatment planning could benefit from a personalized assessment of dental maturation versus skeletal facial growth, but practical tools need to be developed and validated at the population level. From an anthropological perspective, our present study suggests that asynchronous dentofacial development could at least partially explain the frequency of dental crowding in modern populations of children.

## Competing interests

The authors declare that they have no competing interests.

## Authors’ contributions

WYV, JNV, CMS, and JB were responsible for study concept and design. WYV and JD acquired data. JNV performed statistical analyses. WYV, JNV, CMS, PB and JB were involved with interpretation of data. WYV and JNV drafted the manuscript. CMS, JB provided critical revisions to the manuscript for intellectual content. Administrative supervision was provided by PB and JB. All authors read and approved the final manuscript.
